# Anti-Müllerian Hormone Regulation of Synaptic Transmission in the Hippocampus Requires MAPK Signaling and Kv4.2 Potassium Channel Activity

**DOI:** 10.3389/fnins.2021.772251

**Published:** 2021-12-16

**Authors:** Kang Wang, Fuhua Xu, James Maylie, Jing Xu

**Affiliations:** ^1^Department of Obstetrics and Gynecology, School of Medicine, Oregon Health & Science University, Portland, OR, United States; ^2^Division of Reproductive and Developmental Sciences, Oregon National Primate Research Center, Oregon Health & Science University, Beaverton, OR, United States

**Keywords:** anti-Müllerian hormone, hippocampus, electrophysiological recording, Kv4.2, MAPK

## Abstract

Anti-Müllerian hormone (AMH) is a paracrine factor generated peripherally by the gonads to regulate gonadal function in adult mammals. We recently reported that AMH and AMH-specific receptor Anti-Müllerian hormone receptor 2 (AMHR2) are expressed in the hippocampus, and exogenous AMH protein rapidly increased synaptic transmission and long-term synaptic plasticity at the CA3-CA1 synapses. Here we examined the cell-specific expression of AMHR2 and the cellular mechanism of rapid boosting effect of AMH on synaptic transmission in mouse hippocampus. Immunofluorescence staining showed that AMHR2 was specifically expressed in the soma and dendrites of hippocampal pyramidal neurons, but not glial cells. Electrophysiological recordings on acute hippocampal slices showed that AMH did not affect AMPAR-mediated or N-Methyl-D-aspartic acid receptor (NMDAR)-mediated excitatory postsynaptic currents at the CA3-CA1 synapses. The small-conductance Ca^2+^-activated K^+^ channel (SK2) and A-type K^+^ channel (Kv4.2) contribute to shaping excitatory postsynaptic potentials (EPSPs) at the CA3-CA1 synapses. Bath application of apamin to block SK2 did not alter AMH effect on increasing EPSPs, whereas blocking Kv4.2 channel with 4-aminopyridine, or chelating internal Ca^2+^ with BAPTA occluded the action of AMH on boosting EPSPs. Kv4.2 activity is regulated by p38 mitogen-activated kinase (MAPK). Blocking p38 MAPK with SB203580 occluded the effect of AMH on increasing EPSPs. These results show that Kv4.2 channel contributes to the rapid action of AMH on boosting synaptic transmission in a Ca^2+^- and p38 MAPK-dependent manner. Our findings provide functional evidence that AMH enhances synaptic transmission through Kv4.2 channel in the hippocampus, suggesting a possible role of Kv4.2 channel in AMH-regulated neuronal process underlying learning and memory.

## Introduction

Since its first discovery in 1947 by Alfred Jost, numerous studies have expanded the function of anti-Müllerian hormone (AMH) from its eponymous role in sex differentiation to neuroendocrine roles contributing to reproductive fitness and brain circuits (Cate et al., 1986; Munsterberg and Lovell-Badge, 1991; Lee et al., 1996; Lebeurrier et al., 2008; Wang et al., 2009, 2020; Cimino et al., 2016; Tata et al., 2018; Malone et al., 2019). During the stage of fetal development, AMH is secreted by *Sertoli* cells of the male fetus and prevents development of the Müllerian ducts into female reproductive tract (Behringer, 1994). AMH is continuously synthesized and secreted by testes in male to maintain physical characteristics and by ovaries in female to regulate ovarian follicular development during postnatal life (Josso et al., 1998). Interestingly, a rising number of recent studies have reported AMH action, beyond its gonadal function, in the brain circuits to regulate neuronal viability and activity in various brain regions (Cimino et al., 2016; Tata et al., 2018; Malone et al., 2019; Wang et al., 2020).

In a previous study, we reported the expression and direct actions of AMH in the hippocampus. Our findings show that AMH is locally generated in the brain, and rapidly increases synaptic strength and long-term synaptic plasticity (LTP) in the hippocampal CA3-CA1 synapses in mice (Wang et al., 2020). Although these results clearly demonstrate a role of AMH in regulating synaptic transmission in a postsynaptic manner, the underlying mechanism requires further investigation, such as a potential intracellular signaling pathway that delineates the AMH effect on boosting synaptic transmission.

Since AMH is a TGFβ signaling pathway ligand that binds exclusively to Anti-Müllerian hormone receptor 2 (AMHR2), (Rzeszowska et al., 2016) we examined AMHR2 expression in specific cell types, including pyramidal neurons and glia cells, in mouse hippocampal CA1 region. We found that AMHR2 is exclusively expressed in the neuronal soma and dendrites, supporting the direct AMH effect on synaptic responses. The CA1 postsynaptic potentials are mixed postsynaptic ion channel activities, in addition to excitatory AMPAR (α-amino-3-hydroxy-5-methyl-4 isoxazolepropionate acid receptor) and NMDAR (N-Methyl-D-aspartic acid receptor), therefore, increased activities of AMPAR and NMDAR or decreased activities of postsynaptic ion channels, such as the small-conductance Ca^2+^-activated K^+^ channel (SK2) and voltage-gated A-type Kv4.2 channel could lead to the increase in excitatory postsynaptic potential (EPSP) by AMH. Upon glutamate release from presynaptic boutons, the activation of postsynaptic AMPARs depolarizes spines that relieve Mg^2+^ block of glutamate-bound NMDARs, allowing Ca^2+^ influx through NMDARs, which activates synaptic SK2 channels to reduce the amplitude of evoked synaptic potentials (Ngo-Anh et al., 2005; Lin et al., 2008). Synaptic depolarization also activates extrasynaptic Cav2.3 Ca^2+^ channels, the Ca^2+^ influx of which modulates Kv4.2 channel gating and shapes dendritic EPSPs (Wang et al., 2014, 2015). We therefore investigated whether AMPAR, NMDAR or postsynaptic K^+^ channels contribute to the boosting effect of AMH on synaptic transmission. We observed that AMH-enhanced synaptic transmission required intracellular Ca^2+^ and voltage-gated Kv4.2 channels, but not Ca^2+^-activated SK2 channels, in the hippocampus. The dynamic regulation of Kv4.2 channel function requires the interaction of Ca^2+^-sensitive Kv channel-interacting protein (KChIP) to Kv4.2 channel and the direct phosphorylation of the channel pore-forming subunit by p38 mitogen-activated protein kinase (MAPK) (Schrader et al., 2006; Hu et al., 2020). Therefore, we further tested the hypothesis that AMH binding to AMHR2 activates downstream MAPK signaling pathways, which regulates Kv4.2 channel function and alters synaptic transmission. Here we present a novel neuron-specific molecular mechanism that Kv4.2 channel contributes to the rapid action of AMH on boosting synaptic transmission in a Ca^2+^- and p38 MAPK-dependent manner.

## Materials and Methods

### Animals

All animal protocols were approved by the Institutional Animal Care and Use Committee in the Oregon Health & Science University (OHSU). Animals were treated according to the National Institutes of Health guidelines. Because sex differences were not identified for the rapid actions of AMH in regulating the hippocampal synaptic transmission in our previous study, (Wang et al., 2020) male mice were used in the present study. Wild-type adult male CD-1 mice (4 weeks old) were purchased from the OHSU Transgenic Mouse Model Core. Mice were group housed (3–5 per cage) in a temperature- and light-controlled environment in the Division of Comparative Medicine, OHSU. Food and water were provided *ad libitum*.

### Immunofluorescence

Anti-Müllerian hormone receptor 2 protein expression was localized in specific cell types of the hippocampal CA1 region. As described previously, (True et al., 2015) mice (*n* = 3) were sedated with ketamine:xylazine (236:23.6 mg/kg) and perfused transcardially with 4% paraformaldehyde in saline. Brains were collected and post-fixed in 4% paraformaldehyde overnight, followed by 24-h incubation in 30% sucrose, at 4°C. Brains were subsequently frozen on dry ice, cut coronally into 25 μm sections, rinsed in potassium phosphate-buffered saline, and mounted on gelatin-coated glass slides. Immunofluorescence staining was performed as previously described (Wang et al., 2020). Briefly, brain sections were blocked using 2% normal donkey serum (Sigma-Aldrich), and then incubated with primary antibodies at 4°C overnight. In addition to anti-mouse AMHR2 antibody (1:500; MBS1496327; MyBioSource, San Diego, CA, United States), anti-mouse microtubule-associated protein 2 (MAP2; NA10098717; Novus Biologicals, Centennial, CO, United States), glial fibrillary acidic protein (GFAP; AF2594; R&D Systems, Minneapolis, MN, United States), and ionized calcium binding adaptor molecule 1 (IBA1; NB100-1028S; Novus Biologicals, Centennial, CO, United States) antibodies were used as markers for neurons, astrocytes and microglia, respectively. The AMHR2 antibody specificity characterization was performed as reported in our previous study (Wang et al., 2020). Sections were subsequently incubated with Alexa Fluor 488- and Alexa Fluor 594-conjugated secondary antibodies (Invitrogen, Waltham, MA, United States), and DAPI (nuclear staining; Thermo Fisher Scientific). Images were captured using a Revolve microscope (Echo, San Diego, CA, United States).

### Hippocampal Slice Preparation

Mice were anesthetized with isoflurane and rapidly decapitated. The brain was removed and 300 μm slices from the middle of the hippocampus were cut using a vibrating microtome (VT1200S; Leica Instrument, Leitz, Nussloch, Germany) while the brain was immersed in an ice-cold sucrose substituted artificial cerebrospinal fluid (aCSF; control solution) with the following composition (in mM): 119 NaCl, 26 NaHCO_3_, 2.5 KCl, 1 NaH_2_PO_4_, 1 MgCl_2_, 2 CaCl_2_, and 25 dextrose (oxygenated with a carbogen mixture of 95% O_2_ and 5% CO_2_). Slices were held in oxygenated aCSF at 35°C for 30 min, and then at room temperature (22–24°C) for at least 1 h before recording.

### Electrophysiology Recordings

Hippocampal slices were visualized using a fixed-stage, upright microscope (Axioskop; Carl Zeiss, Thornwood, NY, United States) equipped with infrared differential interference contrast optics. The recording chamber was continuously perfused with aCSF equilibrated with 95%O_2_/5%CO_2_ flowing at a rate of 1–2 ml/min. Recording electrodes were pulled from borosilicate pipettes (Sutter Instruments, Novato, CA, United States), and had tip resistances of 2–3.5 MΩ when filled with either K^+^-based or Cs^+^-based internal solution. Patch pipettes for EPSPs were filled with a KMeSO4 internal solution containing 140 mM KMeSO_4_, 10 mM KCl, 10 mM HEPES, 2 mM MgATP, 0.4 mM Na_3_GTP, and 10 mM Tris-phosphocreatine (pH 7.3). For excitatory postsynaptic current (EPSC) recordings in voltage clamp mode, patch pipettes were filled with a CsMeSO_4_ internal solution containing 130 mM CsMeSO_4_, 10 mM CsCl, 10 mM HEPES, 2 mM MgATP, 0.4 mM Na_3_GTP, 10 mM Tris-phosphocreatine, 3.35 mM QX-314, 0.2 mM D600, and 5 mM BAPTA (pH with CsOH to 7.2). Cs^+^ blocks K^+^ channels and QX-314, D600 and BAPTA were added to block voltage gated Na^+^, K^+^, and Ca^2+^ channels and to buffer intracellular Ca^2+^ < 1 nM to block Ca^2+^ activated K^+^ and Cl^–^ channels. Glass stimulating electrodes of approximate resistance of 1 MΩ were filled with aCSF, connected to a Digitimer constant current stimulus isolation unit (AutoMate Scientific, Berkeley, CA, United States), and positioned in the proximal CA1 stratum radiatum, ∼150 μm away from the stratum pyramidale. All whole-cell patch-clamp recordings were made at room temperature (22–24°C). Glutamate release was evoked with synaptic stimulate of 1 ms in duration, delivered once every 20 s.

Recordings were obtained using a Multiclamp 700B amplifier (Molecular Devices, San Jose, CA, United States). Whole-cell recordings were filtered at 3 kHz and digitized at 10 kHz using an ITC- 16 interface (InstruTech, Port Washington, NY, United States), and transferred to a computer using Patchmaster software (Heka, Holliston, MA, United States). Recording usually started 10 min after whole cell formation to all for dialysis. Access resistance (R_access_) and input resistance (R_input_) were monitored throughout the experiments by applying test pulse at the end of each trace (−5 mV in voltage clamp and −25 pA in current clamp). Cells were discarded if R_access_ or R_input_ increased by more than 20%. For current clamp recordings the membrane potential was held at −65 mV by injecting a bias current. Cells were discarded if the bias current was greater than −100 pA or changed by more than 25 pA.

Anti-Müllerian Hormone treatment was achieved by bath application of 0.4 nM recombinant human AMH protein (rhAMH; 1737-MS, R&D Systems, Inc.), as previously described (Wang et al., 2020). For experiments using apamin to block SK2 channels, 4-AP to block Kv4.2 channels or SB203580 to block MAPK, slices incubated in for at least 1 h in the inhibitor before adding AMH. For experiments where BAPTA or 4-AP were added to the patch pipette KMeSO4 internal solution, recordings started > 15 min after whole cell formation to all for dialysis. All experiments were performed in the presence of 5 μM SR95531 and 2.5 μM CGP55845 (Tocris Bioscience) to suppress inhibitory synaptic transmission.

### Statistical Analyses

Voltage traces were analyzed using custom macros written in Igor Pro (WaveMetrics, Tigard, OR, United States). The average of 15 traces before and 20′ after addition of rhAMH was used to calculate the average EPSC or EPSP response. Changes in peak responses are presented as mean ± SEM, and compared statistically using paired two-sample Student’s *t*-test for whole-cell EPSP and EPSC recordings. Ensemble average was compared statistically using a non-parametric Wilcoxon-Mann-Whitney two-sample rank test. *P* < 0.05 was accepted as statistically significant.

## Results

### Anti-Müllerian Hormone Receptor 2 Is Exclusively Expressed by Pyramidal Neurons, but Not Glial Cells, in the Mouse Hippocampal CA1 Region

**FIGURE 1:**
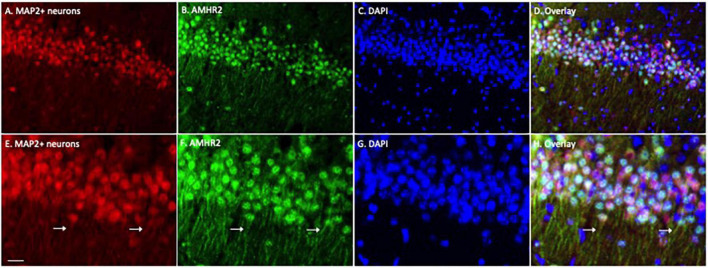
Anti-Müllerian hormone receptor 2 is expressed in the CA1 pyramidal neurons of mouse hippocampus. Representative coronal section was fluorescent-labeled for microtubule-associated protein 2 (MAP2; red; **A,E**), AMHR2 (green; **B,F**), and DAPI (nuclear staining; blue; **C,G**). MAP2 and AMHR2 proteins are co-localized in neurons **(D,H)**. Arrows point to the representative dendrites of CA1 neurons. Scale bar = 40 μm in panel **(A–D)** and 10 μm in panel **(E–H)** (high magnification images).

We first examined the expression of AMHR2 protein in typical cell populations of the adult mouse hippocampus by immunofluorescence staining. Positive immunostaining of MAP2 (red; representatives in Figures 1A,E) and AMHR2 (green; representatives in Figures 1B,F) proteins were co-localized in CA1 pyramidal neurons of the hippocampus (nuclear staining, representatives in Figures 1C,G; overlay, representatives in Figures 1D,H). Both the soma and dendrites (arrows in Figures 1E,F,H) of neurons were MAP2- and AMHR2-positive.

**FIGURE 2:**
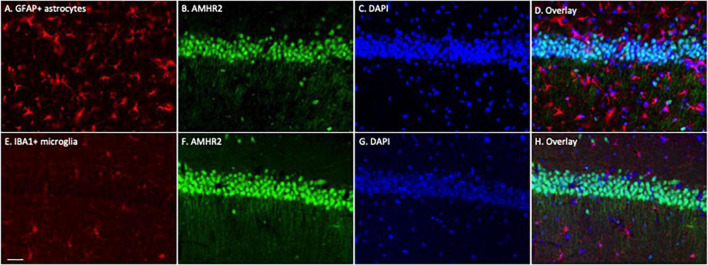
Astrocytes and microglia in the mouse hippocampus do not express AMHR2. Representative coronal sections were fluorescent-labeled for glial fibrillary acidic protein (GFAP; red; **A**), ionized calcium binding adaptor molecule 1 (IBA1; red; **E**), AMHR2 (green; **B,F**), and DAPI (nuclear staining; blue; **C,G**). AMHR2 protein is not co-localized with GFAP **(D)** or IBA1 **(H)**. Scale bar = 40 μm.

To determine whether astrocytes or microglia express AMHR2, additional immunofluorescence staining was performed on the hippocampal sections. Positive immunostaining of GFAP (red; representatives in Figure 2A) and IBA1 (red; representatives in Figure 2E) were detected in astrocytes and microglia, respectively. The staining was not overlapped with AMHR2 signal (green; representative in Figures 2B,F) that was contained in neurons (nuclear staining, representatives in Figures 2C,G; overlay, representative in Figures 2D,H). These results demonstrate that AMHR2 is exclusively expressed by pyramidal neurons, but not astrocytes or microglia, in the hippocampal CA1 region.

### AMPAR and N-Methyl-D-Aspartic Acid Receptor Do Not Contribute to the Increase in Synaptic Transmission by Anti-Müllerian Hormone

**FIGURE 3:**
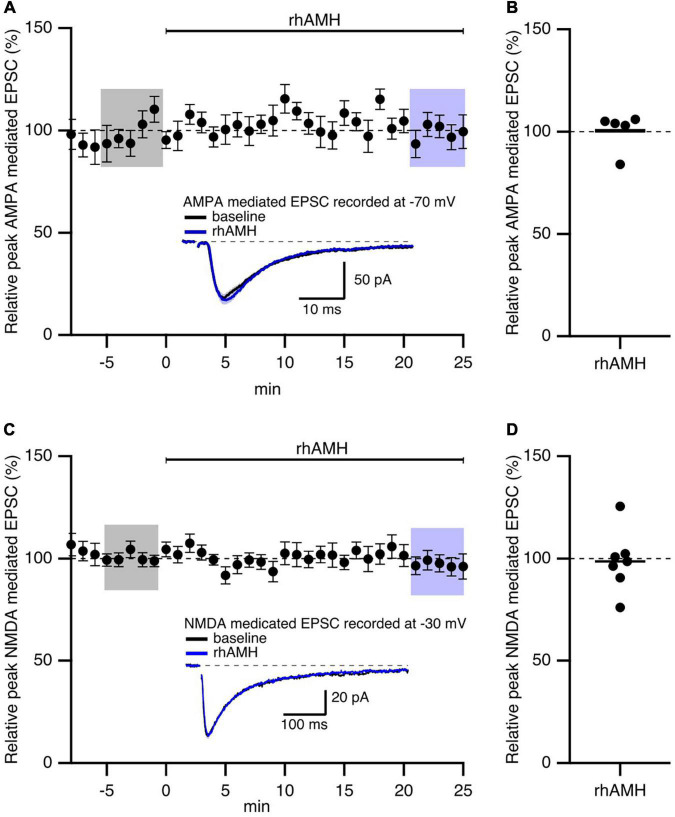
Anti-Müllerian hormone does not affect AMPAR- or NMDAR-mediated EPSCs in mouse hippocampus. Summary time course plots of normalized EPSC amplitudes of hippocampal slices with bath application of 0.4 nM recombinant human AMH protein (rhAMH) at 0–25 min. rhAMH addition did not increase AMPAR-mediated EPSC in the presence of 50 μM D-AP5 **(A)** or NMDAR-mediated EPSC in the presence of 25 μM CNQX **(C)**. Data are binned into 1 min intervalsm mean ± SEM. The current traces used for averaging were taken at time points highlighted with gray and blue shadings. Representative current traces (average of 15 EPSCs) were acquired in the control solution (baseline, black) and in the presence of rhAMH (blue) for individual neurons. **(B,D)** Summary scatterplots of normalized EPSC amplitude.

In general, both presynaptic and postsynaptic components contribute to the responses to synaptic stimulation. Previously, we showed that AMH-induced enhancement of EPSPs was not mediated by an increase in glutamate release at Schaffer collateral-to-CA1 synapses (Wang et al., 2020). To determine the contribution of postsynaptic AMPAR and NMDAR on mediating AMH boosting of synaptic transmission, evoked EPSCs were measured in voltage clamp using a Cs^+^-based internal solution to block postsynaptic K^+^ channels (Wang et al., 2014). D600 (0.2 mM), QX314 (3.35 mM), and BAPTA (5 mM) were added to the pipette solution to block postsynaptic Ca^2+^ channels, Na^+^ channels, and Ca^2+^-activated conductances, respectively. AMPAR-mediated EPSCs were isolated in voltage clamp at −70 mV when D-AP5 (50 μM) was added to the bath solution to block NMDARs. NMDAR-mediated EPSCs were isolated in voltage clamp at −30 mV when CNQX (25 μM) was added to the bath solution to block AMPARs. Under these conditions, rhAMH did not change the AMPAR-mediated EPSCs at −70 mV (100.5 ± 4.1%; 5 slices from 5 mice, *p* = 0.4) (Figures 3A,B) or the NMDAR-mediated EPSCs at −30 mV (98.6 ± 5.6%; 7 slices from 4 mice, *p* = 0.7) (Figures 3C,D).

These results show that the increase in synaptic transmission by AMH is not mediated by AMPARs or NMDARs.

### The Boosting Effect of Anti-Müllerian Hormone Requires Increased Intracellular Ca^2+^ Mobilization

**FIGURE 4:**
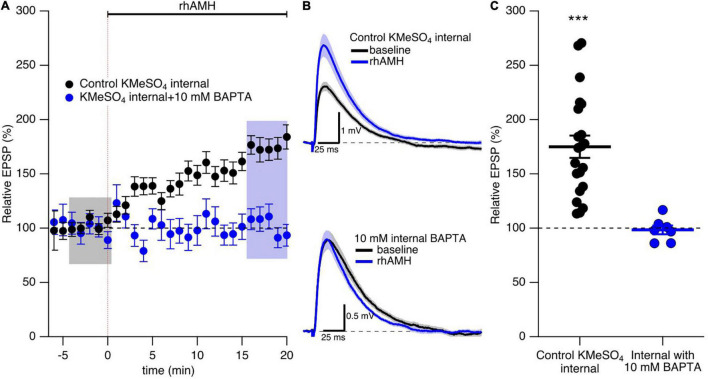
The boosting effect of anti-Müllerian hormone (AMH) requires increased intracellular Ca^2+^ mobilization. **(A)** Summary time course plots of evoked EPSP amplitudes with bath application of 0.4 nM rhAMH at 0–20 min in control KMeSO_4_ internal solution (black symbols) and KMeSO_4_ internal containing 10 mM BAPTA (blue symbols). rhAMH addition increased EPSPs in control internal (*p* < 0.0001) but did not increase EPSPs when intracellular Ca^2+^ was chelated by 10 mM BAPTA (*p* = 0.44). Data are binned into 1 min intervals, mean ± SEM. **(B)** Representative current traces (average of 15 EPSPs) were acquired in the control solution (baseline, black) and in the presence of rhAMH (blue) for individual neurons in control KMeSO_4_ internal (upper panel) and 10 mM BAPTA containing KMeSO_4_ internal (lower panel). **(C)** Summary scatterplot of normalized EPSP amplitude. Bars represent mean ± SEM. *** *p* < 0.0001.

As previously reported (Wang et al., 2020) and reproduced here, bath application of rhAMH increased the peak EPSP to 175% ± 10% (*n* = 21, *p* < 0.0001) of baseline (Figures 4A–C). During synaptic transmission at the CA3-CA1 neurons, Ca^2+^ flows into the spine head through NMDARs and R-type Ca^2+^ channels to increase intracellular Ca^2+^ transients (Sabatini et al., 2002; Ngo-Anh et al., 2005; Higley and Sabatini, 2012). To test whether an increase in Ca^2+^ mobilization is required for AMH boosting of EPSPs, BAPTA (10 mM) was included in the KMeSO_4_ internal patch pipette solution. Under these conditions, rhAMH no longer increased EPSPs (98.3 ± 4.0, *n* = 7 slices from 5 mice, *p* = 0.44) (Figures 4A–C). Therefore, an increase in Ca^2+^ mobilization is necessary for the boosting of EPSPs by AMH.

### Anti-Müllerian Hormone Boosting of Synaptic Responses Does Not Require SK2 Channels

**FIGURE 5:**
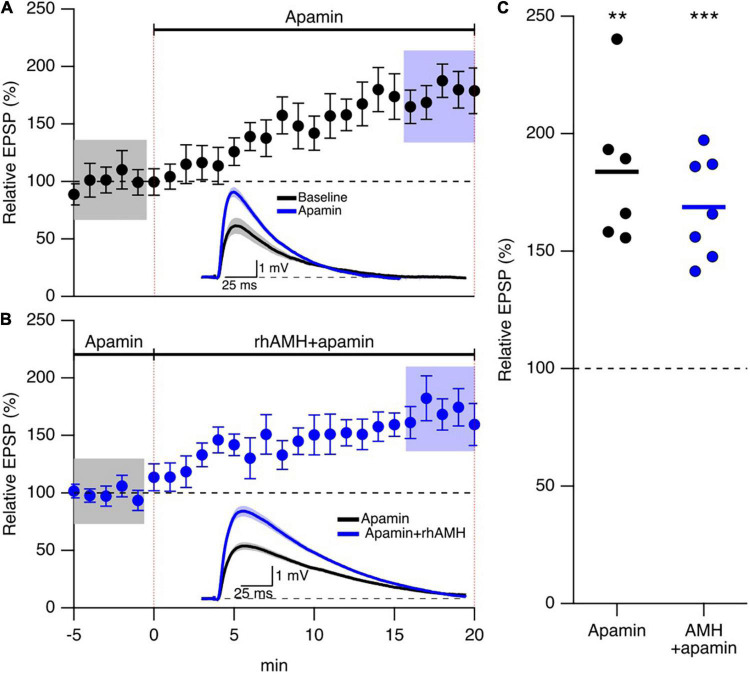
Anti-Müllerian hormone boosting of synaptic responses does not require SK2 channels. **(A)** Summary time course plots of normalized evoked EPSP amplitudes in control aCSF and bath application of 100 nm apamin at 0–20 min. Data are binned into 1 min intervals, mean ± SEM. The voltage traces used for averaging were taken at time points highlighted with gray and blue shadings. Representative voltage traces (average of 15 EPSPs) were acquired in the absence (black) and presence (blue) of rhAMH for individual neurons. **(B)** Summary time course plots of normalized evoked EPSP amplitudes in the presence of 100 nM apamin and bath application of 0.4 nM rhAMH at 0–20 min. Apamin did not occlude the increase in EPSP by rhAMH. Data are binned into 1 min intervals, mean ± SEM. The voltage traces used for averaging were taken at time points highlighted with gray and blue shadings. Representative voltage traces (average of 15 EPSPs) were acquired in the absence (black) and presence (blue) of rhAMH for individual neurons. **(C)** Summary scatterplot of relative increase in EPSP amplitude by apamin (black symbols) and by rhAMH in apamin (blue symbols), ** *p* < 0.01, *** *p* < 0.0001.

The observations above raise the question of how increased Ca^2+^ mobilization contributes to the boosting effect of AMH on EPSPs. An increase in spine Ca^2+^ during synaptic transmission activates SK2 channels that repolarizes the membrane potential to reduce EPSPs, and blocking SK2 channels with 100 nM apamin increases evoked EPSPs in an intracellular Ca^2+^ and NMDA dependent manner (Ngo-Anh et al., 2005). Reproduced here, Figures 5A,C shows addition of 100 nM apamin to control bath aCSF increased EPSPs 173 ± 16% (*n* = 5, *p* < 0.05) To determine whether SK2 channels specifically mediate the boosting of EPSPs by AMH, hippocampal slices were treated with apamin (100 nM) throughout the recordings. EPSPs were measured in whole-cell current clamp from CA1 pyramidal neurons. With SK2 channels blocked, rhAMH increased the evoked EPSPs (162.5 ± 19.1%; 6 slices from 3 mice, *p* = 0.0001) (Figures 5B,C). The rhAMH boosting of EPSPs in control aCSF (175% ± 10% *n* = 21, Figure 4) was not different from the rhAMH boosting of EPSPs in the presence of apamin from Figure 5 (*p* = 0.58, non-parametric Wilcoxon-Mann-Whitney two-sample rank test). Therefore, SK2 channels do not contribute to the boosting of EPSPs by AMH.

### Anti-Müllerian Hormone Boosting of Synaptic Responses Requires Kv4.2-Containing Channels

**FIGURE 6:**
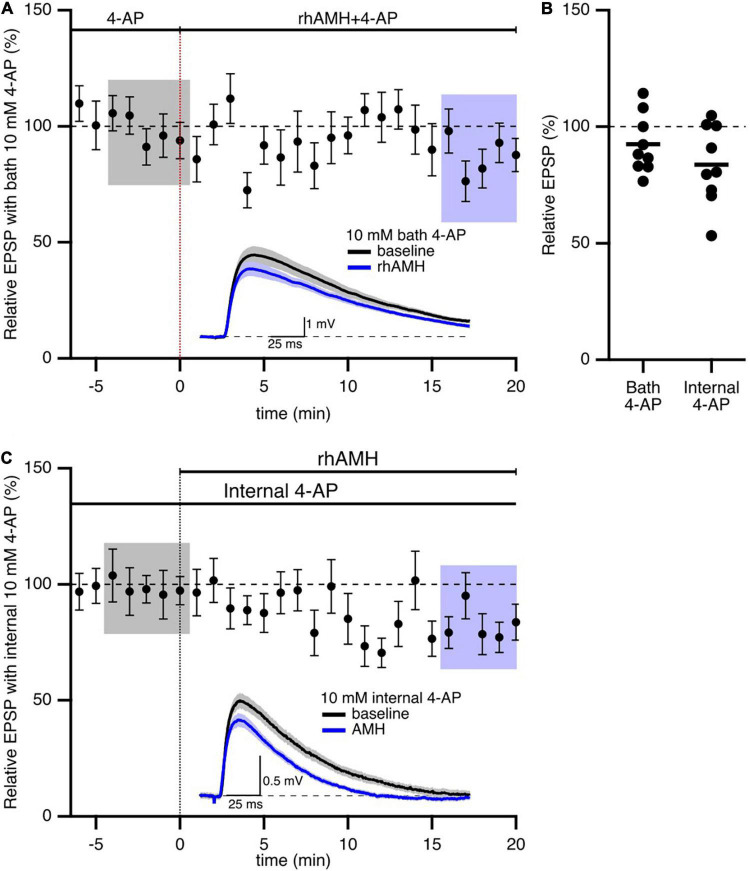
Anti-Müllerian hormone boosting of synaptic responses requires the 4-aminopyridine (4-AP)-sensitive, Kv4.2-containing channels. **(A)** Summary time course plots of normalized evoked EPSP amplitudes in the presence of 10 mM 4-AP in the bath and bath application of 0.4 nM rhAMH at 0–20 min. rhAMH addition did not increase EPSPs in the presence of 4-AP. Data are binned into 1 min intervals, mean ± SEM. Representative voltage traces (average of 15 EPSPs) were acquired in the absence (black) and presence (blue) of rhAMH for individual neurons. **(B)** Summary scatterplot of normalized EPSP amplitude. **(C)** Summary time course and representative traces in the presence of 10 mM 4-AP in the patch pipette internal solution. Following whole cell formation cells were dialyzed for 20 min before evoking EPSPs.

In addition to SK2 channels, the 4-aminopyridine (4-AP)-sensitive A-type Kv4.2 K^+^ channel has significant influence on synaptic transmission at the CA3-CA1 synapses. A-type K^+^ channels containing Kv4.2 subunits are expressed in dendritic spines on CA1 pyramidal neurons (Chen et al., 2006; Kim et al., 2007). Ca^2+^ entry through extra-synaptic Cav2.3 R-type Ca^2+^ channels modulates Kv4.2-containing channel gating, and contributes to repolarizing the spines (Wang et al., 2014). To test whether AMH boosts EPSPs *via* effects on Kv4.2 channels, we obtained baseline recordings in the presence of 4-AP (10 mM) prior to rhAMH application. In this case, rhAMH did not boost EPSPs (89.5 ± 4.8%; 10 slices from 7 mice, *p* = 0.54) (Figures 6A,B). In addition, EPSPs were measured with KMeSO4 internal solution containing 4-AP (10 mM), and bath application of rhAMH did not boost the amplitude of evoked EPSPs (83.7 ± 5.7%, *n* = 9, *p* = 0.27) (Figures 6B,C). These results show that the AMH boosting of EPSPs reveals an underlying coupling between AMH signaling and activation of 4-AP-sensitive Kv4.2 channels.

### p38 Mitogen-Activated Protein Kinase Signaling Is Required for Anti-Müllerian Hormone Boosting of Synaptic Responses

**FIGURE 7:**
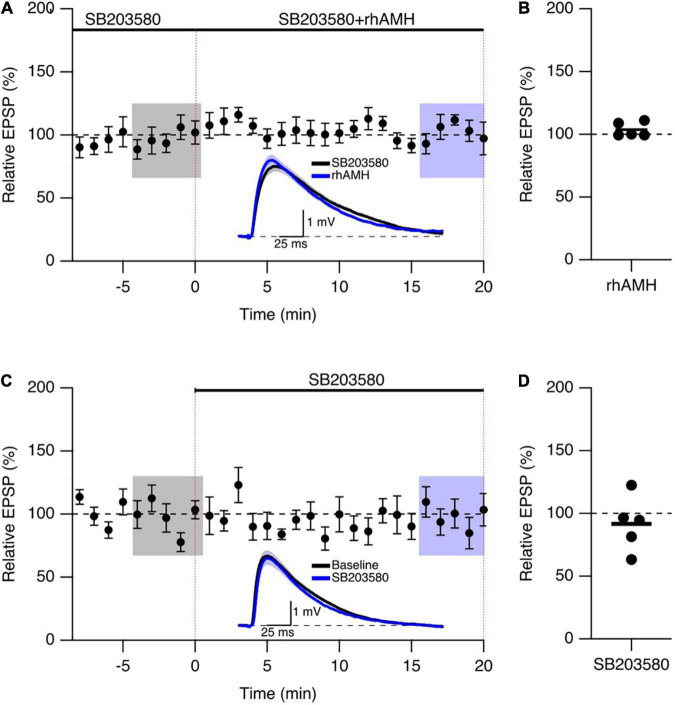
The phosphorylation of Kv4.2-containing channels by p38 mitogen-activated kinase (MAPK) contributes to the AMH boosting of synaptic responses. **(A)** Summary time course plots of evoked EPSP amplitudes in the presence of 10 μM SB203580 at –10–0 min and bath application 0.4 nM rhAMH at 0–20 min. The rhAMH addition did not increase EPSPs in the presence of SB203580. Data are binned into 1 min intervals. Representative voltage traces (average of 15 EPSPs) were acquired in the absence (black) and presence (blue) of rhAMH for individual neurons. **(B)** Summary scatterplot of relative EPSP amplitude during rhAMH addition in the presence of MAPK inhibitor SB203580. **(C)** Summary time course plots of evoked EPSP amplitudes in the presence control aCSF and bath application 10 μM SB203580 at 0–20 min. The SB203580 addition did not increase EPSPs. **(D)** Summary scatterplot of relative EPSP amplitude during SB203580 addition in control aCSF.

We next addressed the mechanism of Kv4.2 channel in regulating the boosting effect of AMH on EPSPs. Since the dynamic regulation of channel function by ERK/MAPK involves the direct phosphorylation of Kv4.2 at T607, (Schrader et al., 2006) and p38 is the primary kinase for the phosphorylation of Kv4.2 at T607 site in mouse hippocampus (Hu et al., 2020). The p38 MAPK inhibitor SB203580 has been used previously to block the induction of Kv4.2 phosphorylation (Hu et al., 2020). To determine whether the boosting of EPSPs by AMH requires p38 MAPK signaling, hippocampal slices were exposed to SB203580 (10 μM) throughout the recording. Bath application of rhAMH did not change the amplitude of evoked EPSPs in the presence of SB203580 (103.7 ± 2.6%, *n* = 5 slices from 3 mice, *p* = 0.31) (Figures 7A,B). Bath application of 10 μM SB203580 by itself did not affect EPSPs (91.6 ± 9.7, *n* = 5, *p* = 0.39) (Figures 7C,D). These results demonstrate that MAPK is required for the direct actions of AMH on boosting EPSPs, suggesting an AMH-AMHR2-MAPK-Kv4.2 signaling pathway in regulating synaptic transmission in the hippocampus.

## Discussion

Using a mouse model, we studied the cell-specific expression of AMHR2 and the mechanism by which AMH increases synaptic transmission in the hippocampus. Our central finding is that AMH binding to AMHR2, the receptor specifically localized in the soma and dendrites of CA1 pyramidal neurons, modulates Kv4.2 gating and increases synaptic transmission, which requires intracellular MAPK signaling. This is the first evidence demonstrating the potential AMH-AMHR2-MAPK-Kv4.2 channel cascade in regulating synaptic transmission in the hippocampus.

The physiological actions of neuronal regulators are always context dependent, i.e., regulating patterns of synaptic connection and types of neurotransmitters in a neuron-specific manner. We previously demonstrated that AMH in the CSF is predominantly generated locally in the central nervous system, (Wang et al., 2020) as AMH produced by the gonads in circulation does not cross the blood-brain barrier (Tata et al., 2018). Our present study determined the ligand-specific receptor AMHR2 protein expression in the soma and dendrites of CA1 neurons, but not in astrocytes or microglia, in the hippocampus. The data suggest that locally produced AMH can elicit a neuron-specific action *via* AMHR2 as a neuronal regulator. AMH binds to pyramidal neurons, and there is no direct involvement of glial cells in AMH-regulated synaptic transmission in the hippocampus. In the present and our previous studies, AMHR2 antibody specificity was characterized by immunohistochemistry using ovarian sections (Wang et al., 2020). Staining using hippocampal sections of *Amhr2*-knockout mice can be performed for additional validation in future studies (Cimino et al., 2016).

Synaptic transmission is the process including the release of presynaptic neurotransmitter and the action of postsynaptic neurotransmitter receptor. We previously reported that AMH increased synaptic transmission in a postsynaptic manner (Wang et al., 2020). Using electrophysiological approaches we showed that AMH did not change AMPAR- or NMDAR-mediated EPSCs. In addition, postsynaptic Ca^2+^ mobilization is required for AMH action on synaptic transmission, as intracellular Ca^2+^ chelation abolished AMH effect on boosting synaptically evoked EPSPs.

We focused on SK2 channels, which are Ca^2+^-activated K^+^ channels, as well as Kv4.2-containing K^+^ channels, which are voltage-activated channels underlying the large A-type current. Both K^+^ channels are largely expressed in the dendrites of CA1 pyramidal neurons in hippocampus. In these neurons, SK2 channels provide a negative feedback of synaptic EPSPs and contribute to synaptic plasticity, (Ngo-Anh et al., 2005; Lin et al., 2008) while A-type currents regulate subthreshold dendritic signals and provide a negative feedback on synaptic EPSPs (Cai et al., 2004; Kim et al., 2005; Wang et al., 2014). Both actions on synaptic transmission require the Ca^2+^ influx either through NMDAR or R-type Ca^2+^ channels in the dendritic spines (Ngo-Anh et al., 2005; Wang et al., 2014). Blocking SK2 channels did not occlude AMH effect on boosting EPSPs suggesting AMH does not modulate SK2 activity. In contrast, blocking Kv4.2-containing channels, either internally or externally, occluded the boosting effect of AMH on synaptic transmission. This clearly demonstrates that the AMH boosting of synaptic responses requires the involvement of Kv4.2-containing channels.

The present study explored the connection between AMH binding to AMHR2 in CA1 pyramidal neurons and the activation of Kv4.2 channels in regulating the synaptic responses. AMHR2 is a serine/threonine kinase receptor, whose action is mediated predominantly by Smads in somatic gonadal cells (di Clemente et al., 2003; Santibanez et al., 2011). There is also evidence suggesting the activation of MAPK pathway upon binding of AMH to AMHR2 in neurons. In a GnRH cell line, blocking MAPK pathway prevented the AMH-dependent induction of cell motility (Malone et al., 2019). Notably, the direct phosphorylation of Kv4.2 at T607 site by ERK/MAPK is involved in regulation of channel function, and p38 MAPK is the primary kinase responsible for the T607 phosphorylation in mouse hippocampus (Schrader et al., 2006; Hu et al., 2020). Our results demonstrated that the inhibition of p38 MAPK abolished AMH effect on increasing synaptically evoked EPSPs, suggesting a signaling cascade of AMH-AMHR2-MAPK-Kv4.2 in the hippocampus. However, it is unclear whether binding of AMH to AMHR2 in hippocampal CA1 neurons directly activate p38 MAPK or *via* additional mediators. Therefore, further studies are needed to delineate the intracellular signaling cascade between AMH-AMHR2 binding and p38 MAPK activation in the hippocampus.

Our previous report demonstrated that AMH increased both synaptic transmission and LTP in the hippocampus (Wang et al., 2020). Synaptic transmission increased by AMH does not account for the increase in LTP, since the increase in synaptic transmission occurred before induction of LTP. Since we demonstrated here that AMH did not affect AMPAR-mediated EPSCs, AMH does not affect the number or the conductance of AMPARs. One LTP model involves the activity-dependent redistribution of Kv4.2 channels, as Kv4.2 internalizes upon synaptically evoked LTP in CA1 neurons of the hippocampus (Kim et al., 2007). Further studies are required to address if the increased synaptic transmission and LTP by AMH involves the trafficking of Kv4.2 channels in the dendritic spines of hippocampus or the increased synaptic transmission by AMH and the ensuing increase in Ca^2+^ influx.

The dynamic regulation of Kv4.2 channel functions requires the direct phosphorylation at T607 site, as well as the interaction between Kv4.2 channel and its auxiliary subunit dipeptidyl peptidase-like 6 and Kv channel-interacting protein, which has EF hand that undergoes conformational changes upon binding of Ca^2+^ (Rhodes et al., 2004; Schrader et al., 2006; Pongs and Schwarz, 2010; Hu et al., 2020). We showed here that AMH-enhanced synaptic transmission requires both intracellular Ca^2+^ mobilization and p38 MAPK signaling. The current study demonstrates the novel finding that Kv4.2 channel contributes to the rapid action of AMH on boosting synaptic transmission in a Ca^2+^- and p38 MAPK-dependent manner, promoting an AMH-AMHR2-MAPK-Kv4.2 signaling cascade in the hippocampus.

## Data Availability Statement

The original contributions presented in the study are included in the article/supplementary material, further inquiries can be directed to the corresponding author/s.

## Ethics Statement

The animal study was reviewed and approved by the Institutional Animal Care and Use Committee in the Oregon Health & Science University (OHSU).

## Author Contributions

JM and JX designed the study. KW and FX performed research. FX, JM, and JX analyzed data. KW, JM, and JX wrote the manuscript. All authors contributed to the article and approved the submitted version.

## Conflict of Interest

The authors declare that the research was conducted in the absence of any commercial or financial relationships that could be construed as a potential conflict of interest.

## Publisher’s Note

All claims expressed in this article are solely those of the authors and do not necessarily represent those of their affiliated organizations, or those of the publisher, the editors and the reviewers. Any product that may be evaluated in this article, or claim that may be made by its manufacturer, is not guaranteed or endorsed by the publisher.
